# Accuracy of body fat percent and adiposity indicators cut off values to detect metabolic risk factors in a sample of Mexican adults

**DOI:** 10.1186/1471-2458-14-341

**Published:** 2014-04-10

**Authors:** Nayeli Macias, Amado D Quezada, Mario Flores, Mauro E Valencia, Edgar Denova-Gutiérrez, Manuel Quiterio-Trenado, Katia Gallegos-Carrillo, Simon Barquera, Jorge Salmerón

**Affiliations:** 1 Center of Research in Nutrition and Health, National Institute of Public Health, Cuernavaca, Mexico; 2 Biology and Chemical Sciences Department, University of Sonora, Hermosillo, Mexico; 3 Center of Medical Sciences Research, Mexico State Autonomous University, Toluca, Mexico; 4 Health Services and Epidemiological Investigation Unit. Cuernavaca Morelos, Mexican Institute of Social Security, Cuernavaca, Mexico; 5 Population Research Center, National Institute of Public Health, Cuernavaca, Mexico

## Abstract

**Background:**

Although body fat percent (BF%) may be used for screening metabolic risk factors, its accuracy compared to BMI and waist circumference is unknown in a Mexican population. We compared the classification accuracy of BF%, BMI and WC for the detection of metabolic risk factors in a sample of Mexican adults; optimized cutoffs as well as sensitivity and specificity at commonly used BF% and BMI international cutoffs were estimated. We also estimated conditional BF% means at BMI international cutoffs.

**Methods:**

We performed a cross-sectional analysis of data on body composition, anthropometry and metabolic risk factors(high glucose, high triglycerides, low HDL cholesterol and hypertension) from 5,100 Mexican men and women. The association between BMI, WC and BF%was evaluated with linear regression models. The BF%, BMI and WC optimal cutoffs for the detection of metabolic risk factors were selected at the point where sensitivity was closest to specificity. Areas under the ROC Curve (AUC) were compared among classifiers using a non-parametric method.

**Results:**

After adjustment for WC, a 1% increase in BMI was associated with a BF% rise of 0.05 percentage points (p.p.) in men (P < 0.05) and 0.25 p.p. in women (P < 0.001). At BMI = 25.0 predicted BF% was 27.6 ± 0.16 (mean ± SE) in men and 41.2 ± 0.07 in women. Estimated BF% cutoffs for detection of metabolic risk factors were close to 30.0 in men and close to 44.0 in women. In men WC had higher AUC than BF% for the classification of all conditions whereas BMI had higher AUC than BF% for the classification of high triglycerides and hypertension. In womenBMI and WC had higher AUC than BF% for the classification of all metabolic risk factors.

**Conclusions:**

BMI and WC were more accurate than BF% for classifying the studied metabolic disorders. International BF% cutoffs had very low specificity and thus produced a high rate of false positives in both sexes.

## Background

Body fat (BF) is a tissue with endocrine and immune functions [1]. When overweight or obesity are present, these characteristics make BF a determinant of metabolic disorders such as insulin resistance and Metabolic syndrome [2]. Overweight and obesity are highly prevalent in the Mexican population [3], and; nationwide representative surveys have shown that 41.6% of Mexican adults have metabolic syndrome [4]. Evaluating BF and its distribution in the Mexican population is thus useful for screening for metabolic risk factors.

Indicators such as BMI and waist circumference (WC) are frequently used to define overweight and obesity, despite the fact that they do not directly measure adiposity. While epidemiological studies have shown these indicators to be useful predictors of non-communicable diseases and mortality risk [5,6], their ability to predict BF has been questioned. Okorodudu et al. [7], in a meta-analysis assessing BMI’s performance in estimating BF%, found that BMI ≥ 30 has good specificity and predictive value for detecting excess adiposity independently of sex, but the authors did not explore the influence of ethnicity on BMI’s performance. This is important since BF% in Caucasians is different from other ethnic groups BF% at the same BMI categories [8].

It has also not been shown which BF% values correspond to the international BMI cutoff points as defined by the World Health Organization (WHO) or the relative performance of BF% for classifying metabolic risk factors in Mexicans. The variations between BF%, BMI and WC in non-Caucasian populations, as well as the high prevalence of metabolic syndrome in Mexicans, highlight the need to assess BF% and the most commonly adiposity indicators cutoff points used for the purpose of the detection of metabolic risk factors in Mexican adults.

## Methods

### Study design and sample

We performed a cross-sectional analysis of baseline data from participants in a cohort study focused on lifestyle and chronic disease (The Health Worker Cohort Study or HWCS) [9]. Briefly, 9467 health care employees from three health and academic institutions in the states of Mexico and Morelos were invited to participate (March 2004-April 2006). Each worker signed informed consent forms, and the study protocol was approved by the Mexican Institute for Social Security (*Instituto Mexicano del Seguro Social*) ethics committee.

The analysis was restricted to subjects aged 20 to 65 years, with body composition and blood sample determinations. Subjects with type-2 diabetes, cancer or other chronic diseases that are known to alter body composition were also excluded. The final analytical sample was composed by 5100 subjects.

### Body composition, anthropometric and biochemical measurements

BF% was determined by DEXA (Lunar DPX-GE, Lunar Radiation; software version 1.35, fast scan mode). Weight was measured to the nearest 100 g (TANITA BC-533). Height was measured while barefoot participants were standing on a flat surface. Waist circumference was measured at the highest point of the iliac crest to the nearest 0.1 cm [10]. Venous blood samples were collected after 8-hours of fasting. Plasma glucose was measured using the glucose oxidize colorimetric method, serum triglyceride concentrations were assayed with a colorimetric method following hydrolysis by lipase, and serum HDL cholesterol was assessed by the clearance method. All biomedical assays were performed with a Selectra XL instrument (Randox, MA USA) in accordance with international proceedings [11]. Trained nurses measured blood pressure with an automatic digital blood pressure monitor while participants were seated with their right arm resting at heart level.

### Variable definitions

The BF% cutoffs chosen to perform sensitivity and specificity comparisons were the values most frequently cited by international scientific literature (BF% ≥ 20 and BF% ≥ 25 for men; BF% ≥ 30 and BF% ≥ 35 for women) [7]. The waist circumference cutoff points used were those recommended by the Mexican Health Ministry (WC ≥ 90 for men and WC ≥ 80 for women) and the NCEP/ATP III revised guidelines (WC ≥ 102 for men and WC ≥ 88 for women) [2]. For both sexes, overweight was defined as BMI ≥ 25 and obesity as BMI ≥ 30 [12].

Metabolic risk factors were defined as high fasting glucose (≥100 mg/dL) [13], high serum triglycerides (≥150 mg/dL) [13], low serum HDL cholesterol (<40 mg/dL in men or <50 mg/dL in women) [2] and high blood pressure (systolic blood pressure >140 mm Hg or diastolic blood pressure >90 mm Hg) [14].

### Statistical analysis

The association between BMI, WC and BF% was evaluated with three regression models estimated for each sex separately using a linear-log specification, which enables to estimate the approximate change in the dependent variable associated with a 1% increase in a given independent variable when its regression coefficient is divided by 100 [15]. This model specification was motivated by looking at scatter plots of the dependent variable vs. each covariate and the pattern described by non parametric (lowess) regressions. In the first model the log of BMI was included as a predictor of BF%, in the second model the log of WC was used as a predictor and the third model incorporated both log BMI and log WC. The percentage of explained variation was compared among models. All analyses were conducted using Stata version 12.1 [16].

The BF%, BMI and WC optimal cutoffs were selected at the point where sensitivity was closest to specificity [17]. Bootstrap percentile 95% confidence intervals were calculated for the optimized cutoffs, sensitivity and specificity,using 1000 replicates [18]. The areas under ROC curves (AUC) were compared among classifiers using a nonparametric method [19].

## Results

### General characteristics of the sample

A total of 3646 women (71.5% of the sample) and 1454 men (28.5% of the sample) were evaluated, and their characteristics are summarized in Table 1. On average, men were heavier, taller, and had larger WC than women. Women had a BF% mean 13.0 percentage points (p.p.) higher than men (*P <* 0.001). Average BF% for men was 24.4, 30.8 and 35.8 for the normal, overweight or obese BMI categories, respectively. For women, BF% was 38.0, 44.2 and 49.3 for the normal, overweight and obese BMI categories. The high glucose and hypertension prevalence were around 25% in men and around 15% in women. In men, the prevalences of high triglycerides and low HDL cholesterol were approximately 60% whereas roughly one third of women had high triglycerides and about 80% presented low HDL cholesterol.

**Table 1:** Characteristics of study subjects

	**Men (n = 1,454)**		**Women (n = 3,646)**	
	**Mean ± SD**	**Median [Min, Max]**	**Mean ± SD**	**Median [Min, Max]**
Age (years)	41.4 ± 10.9	41.0 [20, 65]	41.3 ± 10.9	41.0 [20, 65]
Weight (kg)	76.5 ± 12.7	75.4 [41.9, 161.5]	64.4 ± 11.4	62.8 [40.3, 144.2]
Height (m)	168.7 ± 6.8	169.0 [137, 193]	155.8 ± 6.1	156.0 [136, 180]
***Adiposity indicators***				
BMI (kg/m^2^)	26.8 ± 3.9	26.6 [18.5, 54.4]	26.5 ± 4.5	25.8 [18.5, 52.2]
Waist (cm)	93.0 ± 10.2	92.0 [64, 159]	88.9 ± 11.7	88.0 [58, 152]
Hip (cm)	98.2 ± 7.6	97.0 [80, 146]	100.3 ± 9	99.0 [70, 158]
Waist/hip ratio	0.9 ± 0.1	0.9 [0.7, 1.1]	0.9 ± 0.1	0.9 [0.6, 1.2]
Waist/height ratio	0.6 ± 0.1	0.5 [0.4, 0.9]	0.6 ± 0.1	0.6 [0.4, 1]
BF (%)	29.6 ± 7	29.8 [6.0, 58.2]	42.6 ± 6.3	43.0 [16.7, 65.5]
BFM (kg)	22 ± 8.1	21.3 [2.8, 74.3]	26.5 ± 8.1	25.3 [6.9, 80.8]
***Metabolic indicators***				
Glucose (mg/dL)	96.2 ± 24.8	92.0 [58, 361]	90.3 ± 20.2	88.0 [51, 346]
Triglycerides (mg/dL)	211.7 ± 165.8	175.0 [30, 2490]	143.2 ± 90.6	122.0 [30, 1702]
c-HDL (mg/dL)	37.6 ± 8.6	37.0 [12, 78]	40.0 ± 12.1	39.0 [5, 365]
DBP (mm/Hg)	75.6 ± 10.8	75.0 [42, 124]	71.0 ± 9.6	70.0 [41, 115.3]
SBP (mm/hg)	124.4 ± 13.7	123 [80.7, 235]	114.5 ± 12.6	112 [66, 178]
***Prevalence of metabolic risk factors*** **(%)**				
High glucose^1^	27.2		14.9	
High triglycerides^2^	61.1		34.5	
Low HDL cholesterol^3^	62.2		83.6	
Hypertension^4^	21.5		16.0	

DBP = Diastolic Blood Pressure; SBP = Systolic Blood Pressure.

^1^Glucose ≥ 100 mg/dL.

^2^Triglycerides ≥ 150 mg/dL.

^3^HDL <40 mg/dL for men and <50 mg/dL for women.

^4^SBP >140 mmHg or DBP > 90 mmHg.

All comparisons were statistically significant (P < 0.05) but age.

### BMI and waist circumference associations with body fat percent

In men a BMI increase of 1% was associated with a BF% rise of 0.33 p.p. (Table 2) when using log(BMI) as a single predictor (Model 1). This association weakened when both log(BMI) and log(WC) were included as predictors. In Model 3, a 1% WC increase was associated with an increase of approximately 0.43 p.p. in BF%; once adjusted for WC a 1% increase in BMI related to an increase of 0.05 p.p. in BF%. In men, as a single predictor log(WC) accounts for more variation than log (BMI) and only 0.3 p.p. of explained variation is added when both predictors are used; together they account for 55.9% of BF% variation.

**Table 2:** Models for predicting body fat, by sex

	**Men (n = 1,454)**			**Women (n = 3,646)**		
	**Model 1**	**Model 2**	**Model 3**	**Model 1**	**Model 2**	**Model 3**
Log (BMI)^§^	0.330** (0.011)		0.050* (0.021)	0.295** (0.005)		0.245** (0.007)
Log (WC)^§^		0.483** (0.014)	0.428** (0.028)		0.318** (0.006)	0.082** (0.008)
Constant	-78.7** (3.62)	-189.1** (6.16)	-180.5** (7.43)	-53.8** (1.60)	-100.1** (2.84)	-73.9** (2.70)
R^2^ (%)	43.8	55.6	55.9	56.4	41.9	57.5

^§^Estimates were divided by 100 in order to approximate associated change in the outcome variable for a 1% increase in a given predictor.

Standard errors in parenthesis.

*P < 0.05, **P < 0.001.

All regression coefficients differed by sex (P < 0.01).

In contrast, in women log(BMI) accounts for more variation (R^2^) than log(WC) and its coefficient remains relatively stable when adding log(WC) as a predictor. In the model with both predictors, a 1% increase of BMI was associated with about 0.25 p.p. rise in BF% whereas a 1% increase in WC was associated with approximately 0.08 p.p. rise in BF%. The log(BMI) alone accounts for an amount of variation similar to both predictors jointly (56.4% vs 57.5%).

In Model 1 the BF% conditioned means at BMI = 25 were 27.6 ± 0.16 (mean ± SE) for men and 41.2 ± 0.07 for women; at BMI = 30 they were 34.7 ± 0.20 for men and 47.6 ± 0.10 for women. In Model 2, men’s BF% conditioned means were 28.3 ± 0.13 at WC = 90 and 34.3 ± 0.16 at WC = 102 whereas women’s BF% conditioned means were 39.5 ± 0.11 at WC = 80 and 42.5 ± 0.08 at WC = 88.

### ROC analysis

Optimized BF% cutoff values for the classification of metabolic risks were close to 30.0 in men (Table 3) and close to 44.0 in women (Table 4). In men, estimated BMI cutoffs ranged from 26.3 to 27.2 and WC cutoffs from 92.0 to 94.0. In women, optimized BMI cutoffs ranged from 26.2 to 27.2 and WC cutoffs from 89 to 91, except for HDL < 50.

**Table 3:** Cutoff estimation and AUC comparison between classifiers in adult men

	**Body fat %**			**BMI (kg/m2)**			**Waist (cm)**		
	**Estimated**	**Reference I**	**Reference II**	**Estimated**	**Overweight**	**Obesity**	**Estimated**	**Reference**	**NCEP/ATP III**
***Glucose ≥ 100 mg/dL; n = 1,439***									
Cutoff	30.5 (30.1, 30.9)	20	25	27.0 (26.7, 27.2)	25	30	94.0 (93.0, 95.0)	90	102
Sensitivity	58.3 (55.8, 61.1)	95.1 (92.8, 97.1)	87.0 (83.6, 90.3)	60.9 (57.3, 63.4)	80.6 (76.5, 84.4)	27.4 (22.8, 31.7)	60.6 (57.0, 64.2)	76.0 (71.9, 80.3)	28.9 (24.6, 33.6)
Specificity	58.4 (55.7, 61.0)	10.2 (8.5, 12.2)	25.3 (22.7, 28.2)	60.9 (57.3, 63.5)	37.9 (34.8, 40.9)	85.1 (83.0, 87.3)	59.9 (57.0, 64.4)	43.1 (40.2, 46.1)	85.2 (82.9, 87.5)
AUC	0.611 (0.579, 0.644)			0.639 (0.607, 0.670)			0.647^b^ (0.616, 0.678)		
***Triglycerides ≥ 150 mg/dL; n = 1,439***									
Cutoff	29.6 (29.2, 30.0)	20	25	26.3 (26.1, 26.6)	25	30	92.0 (92.0, 93.0)	90	102
Sensitivity	58.7 (55.5, 61.4)	96.6 (95.3, 97.7)	86.5 (84.2, 88.9)	62.3 (59.7, 65.3)	77.5 (74.7, 80.1)	21.8 (19.1, 24.6)	63.0 (57.1, 65.2)	71.3 (68.5, 74.2)	22.1 (19.5, 24.6)
Specificity	58.8 (55.5, 61.4)	17.1 (14.3, 20.4)	35.2 (30.9, 39.6)	62.3 (59.8, 65.4)	49.1 (45.0, 53.2)	87.3 (84.5, 90.0)	60.0 (57.7, 64.6)	52.5 (48.2, 56.6)	86.8 (83.9, 89.5)
AUC	0.635 (0.605, 0.665)			0.674^a^ (0.645, 0.703)			0.656^b^ (0.627, 0.686)		
***HDL < 40 mg/dL; n = 1,422***									
Cutoff	29.6 (29.2, 30.1)	20	25	26.5 (26.2, 26.7)	25	30	92.0 (92.0, 93.0)	90	102
Sensitivity	55.7 (52.9, 58.2)	93.3 (91.6, 95.0)	82.0 (79.4, 84.6)	56.0 (53.6, 58.8)	72.2 (69.0, 75.1)	21.4 (18.6, 24.1)	59.5 (53.2, 61.6)	68.0 (64.7, 71.1)	22.9 (20.2, 25.5)
Specificity	55.7 (52.9, 58.3)	12.5 (9.9, 15.3)	28.9 (25.1, 32.8)	56.1 (53.5, 58.8)	41.2 (36.9, 45.6)	86.6 (83.8, 89.4)	55.5 (53.6, 60.8)	48.2 (44.3, 52.5)	88.5 (85.8, 91.1)
AUC	0.583 (0.553, 0.614)			0.587 (0.556, 0.617)			0.607^b,c^ (0.577, 0.637)		
***SBP > 140 or DBP > 90 mmHg; n = 1,439***									
Cutoff	30.4 (29.9, 30.8)	20	25	27.2 (27.0, 27.5)	25	30	94.0 (94.0, 95.0)	90	102
Sensitivity	55.7 (52.9, 58.6)	94.8 (92.2, 97.1)	84.1 (80.3, 87.7)	63.4 (60.2, 66.0)	85.4 (81.6, 89.3)	31.7 (25.6, 37.5)	63.8 (57.6, 65.4)	77.3 (72.5, 81.9)	31.1 (26.2, 35.9)
Specificity	55.7 (53.0, 58.6)	9.8 (8.1, 11.6)	24.1 (21.5, 26.5)	63.3 (60.3, 66.1)	38.3 (35.8, 41.2)	85.4 (83.3, 87.3)	59.6 (57.6, 64.8)	42.6 (39.7, 45.3)	84.7 (82.6, 86.6)
AUC	0.578 (0.540, 0.604)			0.680^a^ (0.635, 0.695)			0.650^b, c^ (0.602, 0.664)		

Sensitivity and Specificity expressed as percentages. Bootstrap percentile 95% confidence intervals in parentheses for optimized cutoff, sensitivity and specificity. Asymptotic normal 95% confidence intervals in parenthesis for the AUC. ^a^p<0.05 BMI vs Body fat AUC ^b^p<0.05 Waist vs Body fat AUC ^c^p<0.05 Waist vs BMI AUC. DBP = Diastolic Blood Pressure; SBP = Systolic Blood Pressure.

**Table 4:** Cutoff estimation and AUC comparison between classifiers in adult women

	**Body fat %**			**BMI (kg/m2)**			**Waist (cm)**		
	**Estimated**	**Reference I**	**Reference II**	**Estimated**	**Overweight**	**Obesity**	**Estimated**	**Reference**	**NCEP/ATP III**
***Glucose ≥ 100 mg/dL; n = 3,625***									
Cutoff	44.0 (43.8, 44.4)	30	35	27.2 (26.8, 27.4)	25	30	91.0 (90.0, 91.0)	80	88
Sensitivity	60.9 (58.8, 63.1)	99.6 (99.1, 100.0)	94.8 (92.9, 96.6)	66.4 (64.1, 68.6)	84.0 (81.0, 87.1)	41.2 (36.8, 45.5)	62.3 (60.9, 66.8)	93.1 (90.9, 95.2)	74.6 (71.1, 78.1)
Specificity	60.9 (58.8, 63.0)	3.8 (3.1, 4.5)	13.3 (12.1, 14.5)	66.4 (64.2, 68.7)	46.1 (44.3, 47.8)	85.2 (84.0, 86.5)	65.6 (60.6, 66.9)	23.8 (22.3, 25.2)	54.0 (52.2, 55.7)
AUC	0.645 (0.620, .669)			0.723^a^ (0.700, 0.745)			0.703^b, c^ (0.679,0.726)		
***Triglycerides ≥ 150 mg/dL; n = 3,627***									
Cutoff	43.3 (43.1, 43.5)	30	35	26.2 (26.0, 26.4)	25	30	89.0 (88.0, 89.0)	80	88
Sensitivity	56.0 (54.4, 58.0)	98.6 (97.9, 99.2)	93.2 (91.8, 94.5)	61.0 (59.4, 62.7)	73.8 (71.3, 76.1)	26.7 (24.1, 29.3)	58.4 (56.4, 61.3)	89.1 (87.5, 90.8)	62.2 (59.5, 64.9)
Specificity	56.0 (54.3, 58.0)	4.3 (3.5, 5.1)	14.9 (13.4, 16.3)	61.1 (59.3, 62.8)	49.7 (47.8, 51.9)	85.4 (84.1, 86.8)	60.2 (56.2, 62.0)	26.8 (25.2, 28.6)	55.9 (54.0, 57.9)
AUC	0.584 (0.565, 0.603)			0.652^a^ (0.634, 0.671)			0.633^b, c^ (0.615, 0.651)		
***HDL < 50 mg/dL; n = 3,614***									
Cutoff	42.3 (42.0, 42.7)	30	35	25.3 (25.1, 25.5)	25	30	86.0 (86.0, 87.0)	80	88
Sensitivity	56.2 (53.9, 58.4)	97.1 (96.4, 97.7)	88.8 (87.6, 89.9)	57.6 (55.4, 59.6)	60.9 (59.2, 62.5)	20.2 (18.8, 21.7)	60.9 (55.8, 62.0)	81.4 (80.0, 82.8)	53.1 (51.3, 54.8)
Specificity	56.2 (53.9, 58.4)	5.2 (3.4, 7.1)	16.7 (13.8, 19.7)	57.6 (55.4, 59.6)	54.4 (50.2, 58.8)	88.9 (86.2, 91.2)	57.4 (56.4, 61.8)	34.8 (31.0, 38.7)	64.3 (60.4, 68.0)
AUC	0.577 (0.552, 0.602)			0.595^a^ (0.570, 0.619)			0.629^b, c^ (0.604, 0.653)		
***SBP > 140 or DBP > 90 mmHg; n = 3,613***									
Cutoff	43.8 (43.5, 44.1)	30	35	26.6 (26.4, 26.9)	25	30	90.0 (89.0, 90.0)	80	88
Sensitivity	58.1 (55.5, 60.1)	98.1 (96.9, 99.1)	91.7 (89.6, 93.8)	60.8 (59.1, 63.1)	78.5 (75.4, 81.8)	34.5 (30.6, 38.3)	58.2 (56.5, 62.4)	89.1 (86.3, 91.7)	66.2 (66.2, 70.4)
Specificity	58.1 (55.5, 60.1)	3.5 (2.9, 4.2)	12.8 (11.7, 14.0)	60.8 (59.1, 63.1)	45.5 (43.8, 47.1)	84.4 (83.1, 85.6)	60.4 (56.2, 62.0)	23.4 (21.9, 24.7)	52.8 (51.0, 54.6)
AUC	0.605 (0.578, 0.625)			0.662^a^ (0.639, 0.684)			0.643^b, c^ (0.613, 0.659)		

Sensitivity and Specificity expressed as percentages. Bootstrap percentile 95% confidence intervals in parentheses for optimized cutoff, sensitivity and specificity. Asymptotic normal 95% confidence intervals in parenthesis for the AUC. ^a^p<0.05 BMI vs Body fat AUC ^b^p<0.05 Waist vs Body fat AUC ^c^p<0.05 Waist vs BMI AUC. DBP = Diastolic Blood Pressure; SBP = Systolic Blood Pressure.

In men, WC showed more discriminatory power (higher AUC) than BF% for the classification of all metabolic risk factors (Table 3) whereas BMI outperformed BF%for the classification of high triglycerides and high blood pressure. Sensitivity and specificity were around 57 at BF% optimized cutoffs. In contrast, BF% reference cutoff of 20 showed a very high sensitivity (>90) but low specificity (<20), similarly, the second reference at BF% = 25 showed high sensibility and low specificity. In women, BMI had better discriminatory power than both WC and BF% for the classification of all metabolic risk factors (Table 4), except for HDL < 50 classification for which BMI showed a higher AUC than BF but a lower AUC than WC. WC outperformed BF% for the classification of all conditions. At BF% optimized cutoffs sensitivity and specificity were approximately 60 for high glucose and were both between 56 and 58 for classifying the rest of risk factors. Even more markedly than for men, most referenced cutoffs for women had very high sensibility (>97 for BF% = 30.0 and >88 for BF% = 35.0) but very low specificity (<6 for BF = 30.0 and <17 for BF% = 35.0).

In women, BMI cutoff at 25 had high sensibility (>78) and low specificity (<47) for high glucose and hypertension. On the other hand, at the obesity cutoff sensibility was low (<42) and specificity was high (>84) for all metabolic risk factors. Waist cutoff at 80.0 cm had high sensibility (>80) and low specificity (<35) whereas the NCEP/ATP III cutoff had sensibility around 75 and specificity around 55 for classifying high glucose and relatively balanced sensibility and specificity for the rest of risk factors. See Additional files 1 and 2 for the graphical representation of ROC analyses.

## Discussion

The objective of the present study was to evaluate the accuracy of BF%, BMI and WC for detecting metabolic risk factors in Mexican adults. We also predicted BF% points in accordance with WHO definitions of overweight and obesity based on BMI.

In the population under study, the WHO international BMI cutoff point for classifying overweight overestimated cases with metabolic risk factors. In contrast, the obesity BMI cutoff overestimated the proportion of healthy subjects. For women, the WC cutoff points used in health services and by the NCEP/ATP III incorrectly classified a high portion of healthy people as women with metabolic risk factors; on the other hand, for men the NCEP/ATP cutoffs had higher specificity than sensibility.

The AUC values showed that in general BMI and WC were more accurate than BF% for classifying subjects with metabolic risk factors. This result highlight the usefulness of BMI and WC for public health purposes given their higher accuracy and low cost for measurement. In a recent study [20] in which BF% was determined by bioelectrical impedance, BMI also performed better than BF% as metabolic syndrome components classifier. These unexpected results may be due to the measurement error attributable to the determination of BF%. It may be relevant to use more reliable methods to measure body composition such as four compartment models to assess the relationship between body composition and metabolic risk factors and to compare its classification accuracy with respect to BMI.

In regard to DEXA accuracy, some researchers have performed in vitro validation studies. Individual chemical analyses of piglet’s skin, Body Fat Mass (BFM), Fat Free Mass (FFM) and bone showed that DEXA underestimate BFM values. [21] A plausible explanation about this underestimation is the fact that BFM is surrounded by subcutaneous, intramuscular and visceral tissue plus connective tissue, nervous and blood supply system. These components are not considered in DEXA attenuation coefficient used to estimate BFM [21]. Moreover, DEXA validation studies that have used 4 compartment models as gold standard in healthy adults found that BFM obtained with DEXA underestimate the values obtained with method 4 Compartment model. The DEXA bias was not necessarily attributable to variations in fat-free mass hydration and instead the bias may be related to differences in anterior-posterior tissue thickness [22]. In concordance with these findings, other authors have indicated that body size may bias BF obtained with DEXA through “the effect of tissue depth; with increasing tissue depth associated with greater bias” [23]. On the other hand, some studies have demonstrated that Hispanics have more hepatic fat than other ethnic groups. Such differences maintain their significance after BMI adjustment. Moreover, Hispanics have a higher deep subcutaneous vs deep visceral fat tissue ratio than other groups [24]. The lowest AUC values for DEXA obtained in the present study may be explained by the induced error of measurement in relation to this method disability to detect deep BFM compartments. Although we recognize DEXA limitations to detect BFM, it is important to report body composition relationship with cardiovascular risk factors in our population. In the authors knowledge this is one of the few studies that may exemplify the absence of appropriate BF% cutoffs to identify people with cardiovascular risk in Latino American groups. There is currently no consensus about the use of BF% to define overweight and obesity as there is for using WHO BMI categories and WC in the Mexican population.

In the present study, means of BF% at the overweight BMI category were 30.8 for men and 44.2 for women. At the obesity BMI category BF% means were 35.8 for men and 49.3 for women. In a comparison of different ethnic groups, Gallagher et al. [8] found that African Americans had lower BF% predicted values than White Americans at the same BMI categories and young adult Asians had higher BF% predicted values in comparison with White Americans. Our BF% estimates at BMI categories were higher than those reported for these three ethnicities in Gallagher’s study. These results underscore the relevance of exploring the relationship between BF%, BMI and illness risk in different ethnic populations. Our findings suggest that ethnic differences may cause significant variation in body composition which appears to render the use of international BF% cutoffs inappropriate in the Mexican population.

Okorodudu et al., performed a meta-analysis evaluating BMI’s performance in detecting BF% [7]. They found that international BMI categories used for determining obesity have a high specificity and low sensitivity for identifying adiposity. This means that the BMI obesity cutoff does not adequately identify people with BF% excess.

Our estimated WC cutoff values for men were lower than 102 cm, value purposed by the NCEP/ATPIII initiative. For women, except for HDL < 50, WC cutoff values were greater than that recommended by NCEP/ATPIII and the one currently used for Mexican population. The sensitivity and specificity that we found for WC indicated that WC can be used to classify metabolic risk factors in Mexican men. In contrast, our results suggest that the election of WC cutoff points for women should be determined by the metabolic risk factor that will be evaluated.

Lahmann *et al.*[25] evaluated the association between adiposity measures and causes of mortality in Swedish middle-aged and older men and women. For women and men who were similar in age to the population evaluated by the present study, BF% of 35.0 and 25.0 respectively, and the highest quintile of WC were associated with the highest mortality risk. Although these cutoff values coincide with internationally most used BF% cutoffs, our results highlight the necessity of validating such cutoffs in different ethnic groups. Similarly, Joseph et al. [26] evaluated BF% cutoff values in Asian Indians to identify their risk of presenting cardiovascular factors. They found that 25.5 and 38.0 BF% were adequate to predict cardiovascular risk, for men and women respectively. Values reported for these authors are lower than those that we obtained; these results strengthen the need for population-specific assessment.

BF% cutoff values obtained with ROC analyses for men and women in the present study were higher than the international most used cutoff points in Caucasian populations. At international WHO BMI cutoffs for overweight and obesity, we found higher BF% predicted values than those reported for Mexican-Americans [27], especially in women.

Fernandez JR *et al*. [28] evaluated BF% with DEXA and its association with BMI. They found out that Hispanic American tended to have higher predicted BF% than African Americans and European Americans at BMI > 30 whereas Hispanic American women tended to have higher predicted BF% than the other two ethnic groups at BMI < 30. The aforementioned authors discuss that these differences can be explained in terms of different ancestry background and cultural/lifestyle of Hispanic groups. We agree with the authors in regard to the importance of evaluating the influence of European, African and Indian ancestry on BF% and if cultural-economic-environment factors influence this relationship in different contexts.

Sanchez-Castillo *et al.*[29] using data from a nationwide representative sample found that the BMI cutoff point for hypertension was 26.6 in men and 27.8 in women. Our cutoff for hypertension in men was similar to the one reported by Sanchez-Castillo but differed by about 1.2 points in women.

Men and women in the present study have higher education levels and lower prevalence of metabolic syndrome than the general Mexican population, so our results may not be as representative as those obtained by nationwide surveys; this limits the generalizability of our results. Nonetheless, overall our findings can be useful for evaluating people with ethnic, socioeconomic, and physical characteristics similar to our study participants.

While our approach for obtaining optimal classification cutoffs was focused on accuracy alone, other aspects such as the metabolic risk factors prevalence and the associated treatment costs might be considered as well in future researches. To our knowledge this is the first study to compare the BF% international cutoffs in Mexican population and assess their association with metabolic risk factors. Future studies with population representativeness are required to clarify the relationship between BF% and obesity related indicators of morbidity.

## Conclusions

In the present study BMI and WC showed better accuracy than BF% for detecting metabolic risk factors. BF% international cutoffs had very low specificity and thus a high rate of false positives for both sexes.

## Competing interests

The authors declare that they have no competing interests.

## Authors’ contributions

NM was the main author and responsible of the article; ADQ analyzed data, wrote the paper and had responsibility for its final content; MF, MEV, ED, KG and SB assisted with writing, analysis interpretation and had responsibility for final content. MQ was in charge of laboratory analysis and interpretation of clinical data, JS participated in research design and execution, as well as writing. All authors have read and approved the final manuscript.

## Pre-publication history

The pre-publication history for this paper can be accessed here:

http://www.biomedcentral.com/1471-2458/14/341/prepub

## Supplementary Material

Additional file 1 Body fat, BMI and waist circumference ROC Curves for men, by metabolic risk factor. Click here for file

Additional file 2 Body fat, BMI and waist circumference ROC Curves for women, by metabolic risk factor. Click here for file
