# Supplementary figures and images for: Accuracy of body fat percent and adiposity indicators cut off values to detect metabolic risk factors in a sample of Mexican adults
Source: BMC Public Health. 2014 Apr 10;14:341. doi: 10.1186/1471-2458-14-341 (PMC4108012; doi:10.1186/1471-2458-14-341)

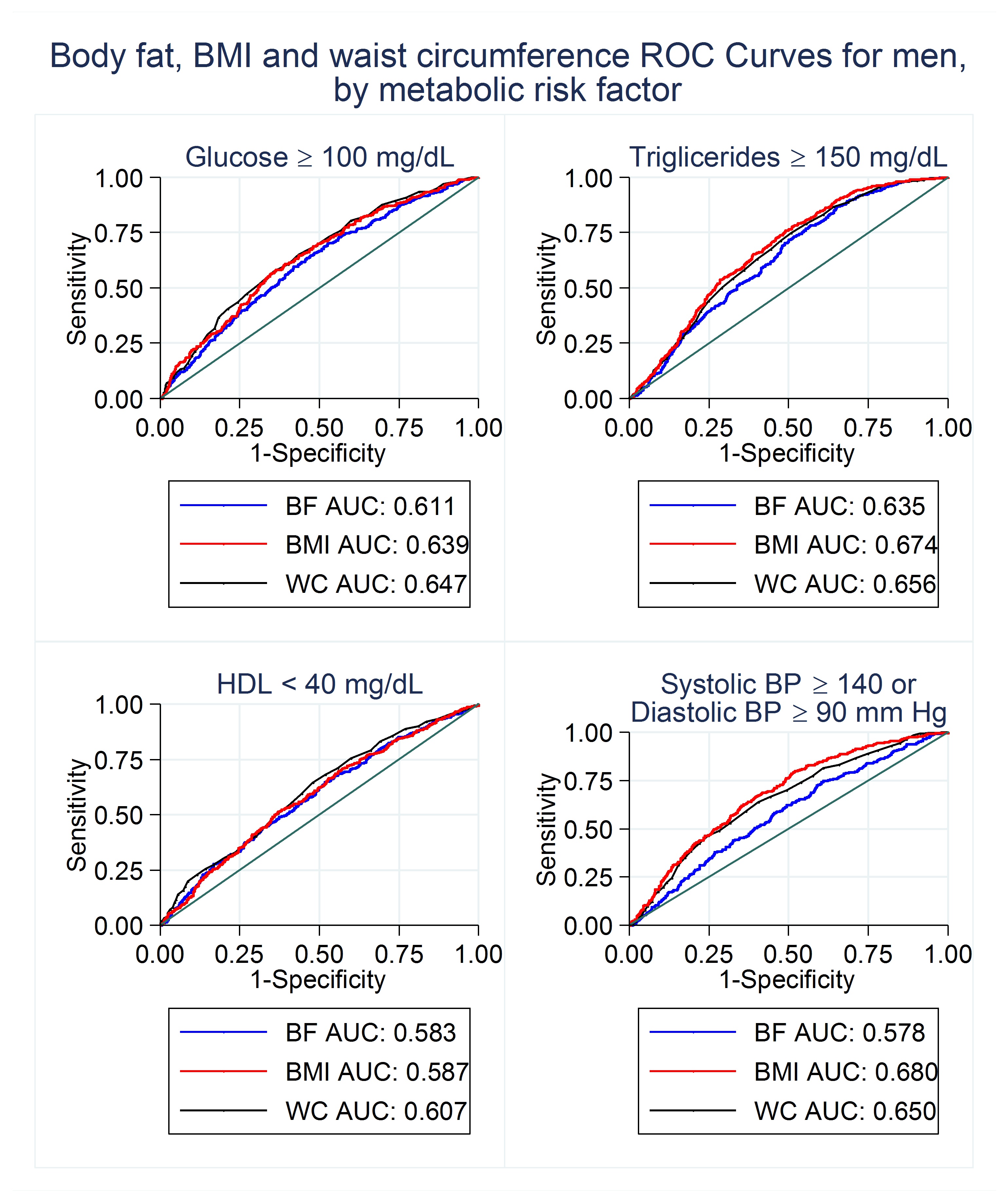

Supplement: Additional file 1 — Body fat, BMI and waist circumference ROC Curves for men, by metabolic risk factor. [file 1471-2458-14-341-S1.jpeg]

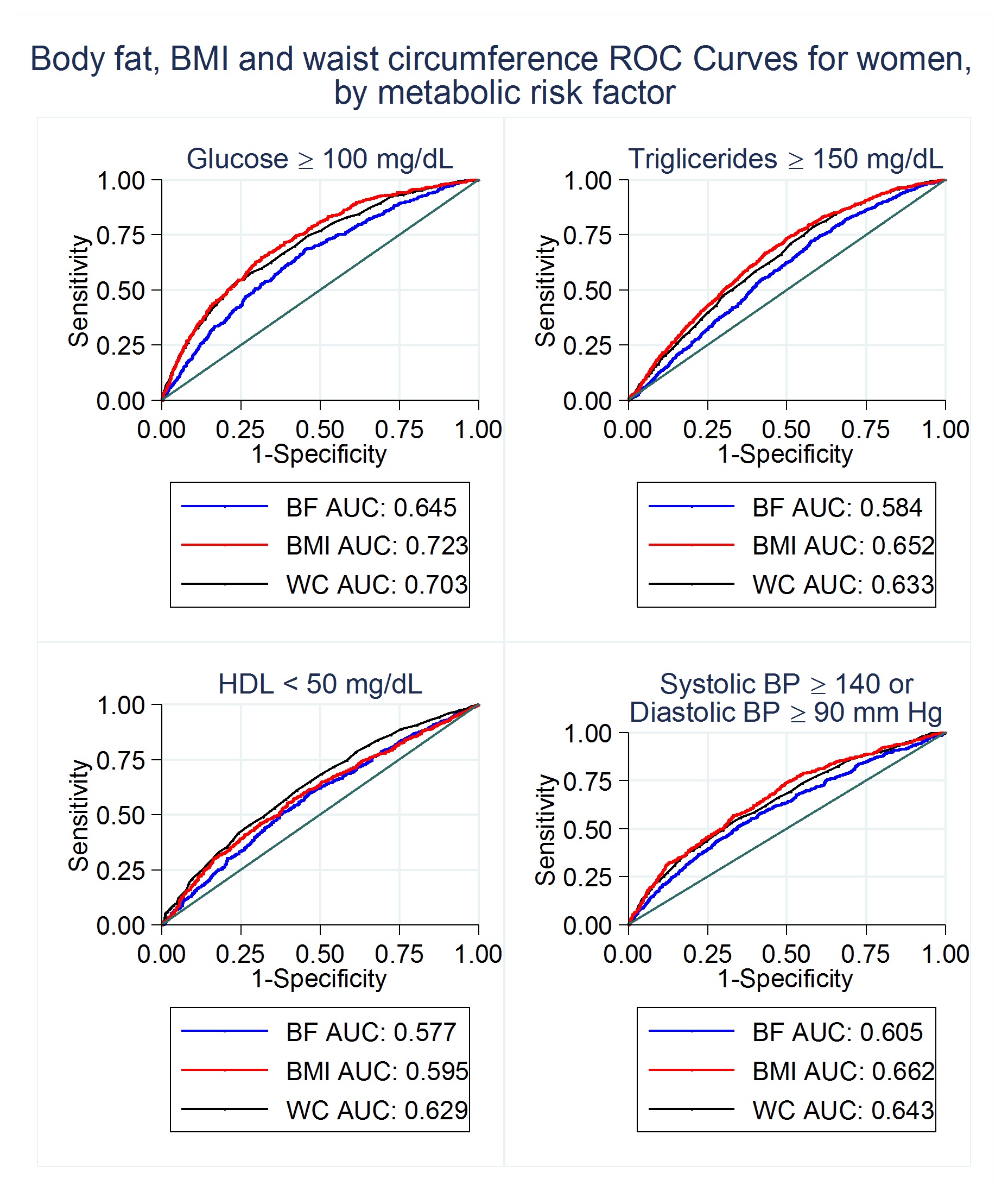

Supplement: Additional file 2 — Body fat, BMI and waist circumference ROC Curves for women, by metabolic risk factor. [file 1471-2458-14-341-S2.jpeg]
